# Default predicted no‐effect target concentrations for antibiotics in the absence of data for the protection against antibiotic resistance and environmental toxicity

**DOI:** 10.1002/ieam.4560

**Published:** 2022-02-21

**Authors:** Jessica Vestel, Daniel J. Caldwell, Joan Tell, Lisa Constantine, Andreas Häner, Jutta Hellstern, Romain Journel, Jim J. Ryan, Tim Swenson, Wei Xei

**Affiliations:** ^1^ Merck & Co. Inc. Kenilworth New Jersey USA; ^2^ Johnson & Johnson New Brunswick New Jersey USA; ^3^ Pfizer Worldwide R&D Groton Connecticut USA; ^4^ F. Hoffmann‐La Roche Ltd. Basel Switzerland; ^5^ Novartis Pharma AG Basel Switzerland; ^6^ Sanofi Gentilly France; ^7^ GlaxoSmithKline Hertfordshire UK; ^8^ TEVA, Parsippany New Jersey USA

**Keywords:** Antibiotic resistance, Environmental risk assessment, PNEC, Wastewater

## Abstract

The pharmaceutical manufacturing industry, via the AMR Industry Alliance, has developed and implemented steps to help minimize the potential impact of pharmaceutical manufacturing on the spread of antimicrobial resistance (AMR). One of these steps was to publish predicted no‐effect concentrations (PNECs) to serve as targets for antibiotic manufacturing wastewater effluent risk assessments aimed to help protect environmental receptors and to mitigate against the spread of antibiotic resistance. Concentrations below which adverse effects in the environment are not expected to occur (PNECs) were first published in 2018 and are updated annually. The current list now stands at 125 antibiotics; however, it is recognized that this list does not encompass all manufactured antibiotics. Therefore, a statistical evaluation of currently available data was conducted and a default PNEC of 0.05 µg/L for antibiotics in the absence of other data was derived. *Integr Environ Assess Manag* 2022;18:863–867. © 2022 Merck, Sanofi, Johnson & Johnson Services, Inc, F.Hoffmann‐La Roche Ltd, Teva Pharmaceuticals, GlaxoSmithKline, Novartis Pharma AG, and Pfizer lnc. *Integrated Environmental Assessment and Management* published by Wiley Periodicals LLC on behalf of Society of Environmental Toxicology & Chemistry (SETAC).

## Key Points

- PNECs were previously published for 125 antibiotics; however, it is recognized that this list does not encompass all manufactured antibiotics.
- A default PNEC value is necessary in order to provide a target for antibiotics with limited data.

## INTRODUCTION

The Wellcome Trust Review on Antimicrobial Resistance recommends setting minimum standards for the manufacturing of antibiotics based on the current state of the science (O'Neill, 2016). The use of appropriate measures based on risk to adequately control the release of antibiotics in manufacturing wastewater effluent remains a priority for the pharmaceutical industry, and is an approach already adopted by many companies (Caldwell et al., 2016; EFPIA, 2021).

The original signatories to the AMR Industry Roadmap, which are now part of the AMR Industry Alliance, made commitments to build and to share common practices addressing manufacturing‐related concerns by specifically focusing on reducing the environmental impact from the production of antibiotics. While the major source of human pharmaceuticals entering into the environment (PIE) is via patient excretion following use of medicine that is taken to prevent, cure, or alleviate a medical condition (particularly from health care facilities), a comparatively smaller contribution to PIE stems from wastewater emissions from industry during the manufacturing of the pharmaceutical ingredients (BIO Intelligence Service, 2013; EFPIA, 2021; Larsson, 2014; Pepper et al., 2018). The environmental concentrations associated with such discharges locally can be much higher than those resulting from usage and excretion (Aga, 2007; Kraupner et al., 2021; Larsson, 2014).

It was identified that inadequate treatment or controls of wastewater effluent from pharmaceutical manufacturing may lead to negative impacts on the local receiving aquatic environment, and, in the case of antibiotics, also may contribute to the development of antibiotic resistance (Bengtsson‐Palme et al., 2018). Therefore, one goal of the AMR Industry Alliance was to reduce production‐related losses such that concentrations in the receiving environment are lower than those likely to result in adverse effects. Toward that end, predicted no‐effect concentrations (PNECs) that are intended to be used as targets for wastewater effluent risk assessments from antibiotic manufacturing were published (Tell et al., 2019). These target PNEC values are compared to predicted or measured concentrations (PEC or MEC, respectively) in the receiving aquatic environment to assess potential risk, which is consistent with current practices (EU WFD, 2018).

The published PNEC targets do not include every antibiotic nor every antibiotic class. Many antibiotics are generic drugs that do not have environmental toxicity data and resources and/or expertise may be limited to develop new PNECs. Therefore, it is necessary to establish default values specifically for antibiotics without data to address ecological receptors in surface water and to minimize the potential for the development of antibiotic resistance in the environment. Controlling wastewater effluents to the default PNEC is expected to result in a significant reduction of antibiotic discharge, especially for antibiotics where specific PNECs have not been defined. The default PNECs may then be applied in a site‐specific manner that considers characteristics of the local situation (e.g., wastewater treatment capabilities, stream flow, location of receptors).

## METHODS

The environmental predicted no‐effect concentration (PNEC‐ENV) and minimum inhibitory predicted no‐effect concentration (PNEC‐MIC) values were taken from PNEC discharge targets published by the AMR Industry Alliance (2021). The following sections detail the methodology used to establish these values.

### PNEC‐ENV

For purposes of the methodology, antibiotics were defined as those active pharmaceutical ingredients (APIs) classified by the WHO Collaborating Centre for Drug Statistics Methodology as per their Anatomical Therapeutic Chemical (ATC) codes. Environmental toxicity data generated for antibiotics in support of regulatory drug approvals in the European Union (EMA European Medicines Agency), as well as other company voluntary programs, were compiled. Data from peer‐reviewed literature were also collected when the studies were deemed to be reliable (e.g., followed OECD guidelines or similar). Studies performed in compliance with the OECD Principles of Good Laboratory Practice were used preferentially.

The PNEC‐ENV values were derived from toxicity endpoint data with an assessment factor applied, consistent with European Guidance (ECHA, 2008; EU WFD, 2018). Since cyanobacteria are considered most sensitive to antibiotics (EMA, 2006; Le Page et al., 2017), data sets were considered complete if cyanobacteria data were developed following the OECD 201 guideline, or equivalent. A PNEC‐ENV was only calculated for APIs with at least one result from a cyanobacteria study.

The lowest chronic no observed effect concentration (NOEC) or 10% effect concentration (EC10) of all species tested for each API was divided by a factor of 10, representing extrapolation to ecosystem effects, to derive a PNEC‐ENV value. In cases where multiple EC10 or NOEC values were available for the same species for the same API, and they were within 1 order of magnitude, the geometric mean of the two values was used to determine the PNEC‐ENV. The geometric mean is used when comparable data on the same end‐point and species are available (ECHA, 2008). For all others, the lowest EC10 or NOEC was used to derive the PNEC‐ENV.

### PNEC‐MIC

Minimum inhibitory concentration (MIC) data from the EUCAST database were compiled by Bengtsson‐Palme and Larsson (2016). The MIC is the lowest concentration of an antibiotic that inhibits 100% of the visible growth of a given strain of bacteria after 24 h of incubation. According to the methodology in Bengtsson‐Palme and Larsson (2016), the 1% lowest observed MICs were identified for each antibiotic and then were adjusted for the number of tested species through modeling. The PNEC‐MIC were then calculated using an assessment factor of 10 to account for differences between MICs and minimal selective concentrations (the lowest concentration of an antibiotic that results in the selection of a resistant mutation in a population and can provide these mutant strains a competitive advantage based on growth rate (Andersson & Hughes, 2014). Of note, methodologies to determine selective effect concentrations of antibiotics in environmental compartments are still being developed and a universally accepted process has not yet been adopted (Murray et al., 2020).

### Percentile estimates

The 5^th^ percentile of all PNEC‐ENV and PNEC‐MIC values were calculated using TIBCO^®^ Spotfire^®^ 10.10.2. Further, 5^th^ percentiles were also calculated for individual antibiotic drug classes (e.g., cephalosporins, macrolides). The 5^th^ percentile was selected in order to identify the PNEC, which would be protective with 95% confidence. The approach of statistical extrapolation is consistent with regulatory guidance, is applicable when more than 15 NOECs are available, and has the advantage of using the whole sensitivity distribution in an ecosystem to derive a PNEC (ECHA, 2008).

## RESULTS

**Figure 1:**
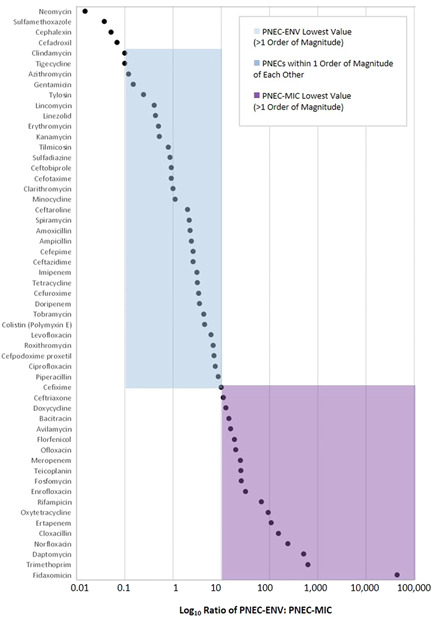
The log_10_ ratio of the PNEC‐ENV to the PNEC‐MIC for each antibiotic in the data set. PNECs were within 1 order of magnitude of each other the majority of the time (60%), with the PNEC‐MIC driving the target for the majority of the remainder (33%). PNECs, predicted no‐effect concentrations; PNEC‐ENV, environmental predicted no‐effect concentration; PNEC‐MIC, minimum inhibitory predicted no‐effect concentration

PNEC values were available for a total of 125 antibiotics, and of these, both PNEC‐ENV and PNEC‐MIC values were available for 55. Where both PNECs were available for the same antibiotic, 60% were within 1 order of magnitude of each other. For the remainder, the PNEC‐MIC tended to drive the overall PNEC more often (32.7%) (Figure 1).

The average and standard deviation of all PNEC‐ENV and PNEC‐MIC values were 5.3 ± 21 µg/L. The calculated 5^th^ percentiles for the PNEC‐ENV and PNEC‐MIC values were 0.05 and 0.06 µg/L, respectively (Table 1). In general, there were not enough antibiotics in each drug class to develop class‐specific default PNEC values.

**TABLE 1:** Fifth percentile estimates for individual antibiotic drug classes and all antibiotics in the data set

ATC drug class	*N*	Fifth percentile estimates	
		PNEC‐ENV (µg/L)	PNEC‐MIC (µg/L)
Aminocyclitol	1	N/A^a^	32.0
Aminoglycoside	8	0.050	0.210
Aminonucleoside	1	31.0	N/A
Amphenicol	2	N/A	1.35
Antifungal	1	N/A	0.250
Antiseptic	1	210	N/A
Antituberculosis agent	5	1.15	0.350
Carbapenem	4	0.420	0.070
Carboxylic acid	1	N/A	0.250
Cephalosporin	26	0.120	0.0600
Cyclic lipopeptide	1	510	1.00
Cyclic peptide	1	115	8.00
Diarylquinolines	1	0.080	N/A
Fluoroquinolone	12	0.660	0.050
Glycopeptide	2	12.9	0.880
Hydrazide	1	N/A	0.130
Imidazole	1	N/A	0.130
Ionophore	1	N/A	0.500
Lincomycin	1	0.100	1.00
Lincosamide	1	0.810	2.00
Macrolactam	1	0.110	N/A
Macrolide	12	0.030	0.040
Monobactam	1	N/A	0.500
Nitrofuran	1	N/A	64.0
Nitroimidazole	2	0.0300	1.00
Novel drug class^b^	1	52.4	2.00
Orthosomycin	1	125	8.00
Oxazolidinone	2	3.22	8.00
Penem	1	N/A	0.0200
Penicillin	11	0.580	0.0900
Phenicol	1	38.0	2.00
Pleuromutilin	2	N/A	0.110
Polymixin	2	0.500	2.00
Polypeptide	1	4.80	N/A
Quinolone	2	N/A	1.28
Steroid antibacterial	1	N/A	0.500
Streptogramin	2	71.1	2.00
Sulfonamide	2	1.13	13.2
Tetracycline	6	0.350	0.600
Trimethoprim	1	312	0.500
All combined	125	0.05	0.06

Abbreviations: ATC, Anatomical Therapeutic Chemical; N/A, not available; PNEC‐ENV, environmental predicted no‐effect concentration; PNEC‐MIC, minimum inhibitory predicted no‐effect concentration.

^a^ PNEC values not derived due to lack of data.

^b^ Novel drug class; antibiotic chemical structure unrelated to other known antibiotics.

## CONCLUSION

Assessing site‐specific risk from manufacturing wastewater effluent has become common practice in the evaluation of pharmaceuticals in the environment. One challenge in conducting the risk assessment is the availability of PNEC values. The AMR Industry Alliance has combined resources to set PNEC targets for antibiotics and to make the PNECs publicly available. However, the list is not comprehensive, and assessors are left with the challenge of what to use in the absence of PNEC data. Based on the above statistical evaluation, antibiotic manufacturers may opt to employ a default value of 0.05 µg/L if the compound belongs to one of the classes studied (Table 1) and when only limited data are available (e.g., no PNEC‐MIC or PNEC‐ENV, limited test or literature data, and read‐across cannot be performed), which is consistent with the recommendations of previously published work (Le Page et al., 2017). As this recommended default value is the lower of the two 5^th^ percentiles calculated for the PNEC‐MIC and the PNEC‐ENV, it is expected to be inclusive of both the PNEC‐MIC and PNEC‐ENV data, and incorporating it into the risk assessment for evaluating wastewater effluent discharges may help to develop improvements in effluent management, to decrease the selection pressure for antibiotic resistance and to protect ecological resources. This default value is considered to be conservative and should be used to minimize antibiotic resistance and environmental toxicity. While the science continues to evolve, more information can be identified on the environmental contribution to antimicrobial resistance (AMR), survival of antibiotic‐resistant genes, and whether or not these genes can be transferred to other bacteria via horizontal gene transfer, ultimately resulting in human health risks (Murray et al., 2020; Pepper et al., 2018).

Continued engagement in scientific discussion with all stakeholders with the aims of expanding the knowledge‐base, developing and improving ways to assess risks, and optimizing strategies to deal with AMR, without compromising patient access to necessary medicines, is fundamental to continuing to develop ways to mitigate against AMR.

## CONFLICT OF INTEREST

The authors are all full‐time employees of international pharmaceutical companies, working as environmental toxicologists, risk assessors, microbiologists, and engineers.

## Supporting information

This article contains online‐only Supporting Information.

The supporting information contains all environmental toxicity data developed or compiled by the authors for each antibiotic in the dataset. The table also includes PNEC‐MIC and PNEC‐ENV values and an explanation of how the PNEC‐ENV was derived.

## Data Availability

Detailed environmental toxicity data from unpublished studies and relevant literature to support the work are available in the supplemental data. Additionally, all data are available by contacting the corresponding author, Jessica Vestel (jessica.vestel@merck.com) or are accessible on the AMR Industry Alliance webpage (https://www.amrindustryalliance.org/).
